# Pressure-Responsive Conductive Poly(vinyl alcohol) Composites Containing Waste Cotton Fibers Biochar

**DOI:** 10.3390/mi13010125

**Published:** 2022-01-13

**Authors:** Mattia Bartoli, Daniele Torsello, Erik Piatti, Mauro Giorcelli, Amelia Carolina Sparavigna, Massimo Rovere, Gianluca Ghigo, Alberto Tagliaferro

**Affiliations:** 1 Center for Sustainable Future Technologies—CSFT@POLITO, Via Livorno 60, 10144 Torino, Italy; 2 Consorzio Interuniversitario Nazionale per la Scienza e Tecnologia dei Materiali (INSTM), Via G. Giusti 9, 50121 Florence, Italy; mauro.giorcelli@polito.it (M.G.); massimo.rovere@polito.it (M.R.); 3 Politecnico di Torino, Department of Applied Science and Technology, C.so Duca degli Abruzzi 24, 10129 Turin, Italy; daniele.torsello@polito.it (D.T.); erik.piatti@polito.it (E.P.); amelia.sparavigna@polito.it (A.C.S.); gianluca.ghigo@polito.it (G.G.); 4 Istituto Nazionale di Fisica Nucleare, Sez. Torino, Via P. Giuria 1, 10125 Turin, Italy; 5 Faculty of Science, University of Ontario Institute of Technology, Oshawa, ON L1G 0C5, Canada

**Keywords:** biochar, PVA, conductive composites, microwave, piezoresistive

## Abstract

The development of responsive composite materials is among the most interesting challenges in contemporary material science and technology. Nevertheless, the use of highly expensive nanostructured fillers has slowed down the spread of these smart materials in several key productive sectors. Here, we propose a new piezoresistive PVA composite containing a cheap, conductive, waste-derived, cotton biochar. We evaluated the electromagnetic properties of the composites under both AC and DC regimes and as a function of applied pressure, showing promisingly high conductivity values by using over 20 wt.% filler loading. We also measured the conductivity of the waste cotton biochar from 20 K up to 350 K observing, for the first time, hopping charge transport in biochar materials.

## 1. Introduction

The advancement in materials science has enabled several new and exciting challenges. Among them, the development of stimuli-responsive materials is very attractive for plenty of technological applications [1,2] ranging from drug delivery [3] to sensors [4] development. The production of sensors is an extensive field that comprises materials responsive to chemical [5], mechanical [6], and electrical [7] input.

Pressure-responsive materials have received significant attention by both academic and industrial communities for biomedical [8], automotive [9], and aerospace [10] utilizations. These materials are generally composed by an inorganic [11,12] or polymeric [13] stretchable/compressible matrix and a dispersed conductive filler. For these applications, the most widely used fillers are carbon-based species, such as carbon nanotubes [14], as well as graphene and graphene-like materials [15]. The amazing performance of carbon fillers is counterbalanced by their high cost [16] and several unsolved production issues [17] that make them unsuitable for large-scale applications.

The needs of high-performance carbon filler, together with affordable costs, opened the way for the use of materials such as biochar. Biochar is the solid waste obtained from the conversion of biomasses under pyrolytic conditions [18]. As reported by Woolf et al. [19], the consolidation of biochar use could represent a significant advance in global warming mitigation. Nevertheless, biochar is mainly used for agricultural and environmental applications only, without exploiting its full potential. As mentioned by Bartoli et al. [20], biochar is a major player in the preparation of conductive elements and composites [21].

Giorcelli et al. [22] produced a piezoresistive device based on an annealed biochar containing silicon composites with remarkable response to pressure. Similarly, Nan et al. [23] produced a reversible pressure-responsive biochar containing a poly(vinyl alcohol) (PVA) composite whose response was affected by filler percentage, thickness, and working temperature. Another approach was reported by Noori et al. [24] with the development of a poly(propylene) composite containing tea biochar. The authors produced a composite material that displayed an irreversible increment of electrical conductivity under pressure. Such materials could also exploit other properties such as the microwave shielding, as reported by several studies [25,26,27]. The combination of pressure-responsive and microwave shielding suggests the use of such materials as multifunctional carbon-based composites for advanced applications.

In this paper, we report on the use of waste cotton fabrics as a source of biochar for the production of pressure-responsive composites. We evaluate the irreversible increment of electrical conductivity of the composites under a wide range of pressures together with their behaviour under microwave irradiation. We also observe, for the first time, hopping charge transport in biochar fibers.

## 2. Materials and Methods

### 2.1. Materials

Waste cotton fibers were recovered from end-life lab coats. They were washed in water and dried at 105 °C for 72 h. PVA in water solution (40 wt.%) was purchased from Giotto Vinilink™ and used without any further purification.

### 2.2. Methods

Waste cotton fibers biochar (WCB) was obtained by pyrolyzing the waste cotton fibers in a tubular furnace (Carbolite TZF 12/65/550, Derbyshire, UK) in nitrogen atmosphere using a heating rate of up to 15 °C/min reaching 1000 °C. The system was kept at 1000 °C for 30 min and cooled down at room temperature in nitrogen atmosphere.

WCB was mechanically grinded for 10 min and dispersed into the PVA matrix according to the procedure reported by Bartoli et al. [28] using a tip ultrasonicator apparatus (Sonics Vibra-cell, Newtown, CT, USA). Ultrasounds were pulsed with cycles of 30 s (20 s pulse, alternating to a pause of 10 s) to avoid an uncontrolled temperature increase. Composite materials were dried in a ventilated oven for 16 h at 70 °C.

The morphology of WCB was investigated from the morphological point of view using both field-emission scanning electron microscopy (FESEM, Zeis SupraTM 40, Oberkochen, Germany) and optical microscopy (Leica M125C, Oberkochen, Germany).

Electric transport measurements on WCB were performed in the four-point van der Pauw configuration by electrically contacting the biochar samples with thin gold wires and conducting silver paste as described in Jagdale et al. [29]. For each van der Pauw configuration, a constant current of 0.1 mA was sourced between two adjacent contacts with a B2912 source measure unit, and the voltage drop occurring across the opposite two contacts was measured with a 34,420 nanovoltmeter (Keysight Technologies, Santa Rosa, CA, USA). Thermoelectric voltages were removed by inverting the sourced current flow within each resistance measurement. The sheet resistance R_s_ was then determined by solving the van der Pauw equation [30], and the DC electrical conductivity as $\sigma =R_{s}^{-1}t^{-1}$, where t≈200 μm is the sample thickness. The temperature dependence of σ was measured by loading the samples in the high-vacuum chamber of an ST-403 pulse-tube cryocooler (Cryomech, Syracuse, NY, USA), cooling the system to the base temperature of 2.7 K, and then quasi-statically warming up the samples to 350 K.

Neat and pyrolyzed cotton fibers were analyzed through FT-IR (Nicolet 5700, Thermoscientific, Waltham, MA, USA) on an attenuated total reflectance (ATR) mode (Smartorbit, Thermoscientific, Waltham, MA, USA) in the range from 500 to 4000 cm^−1^, and through Raman spectroscopy using Renishaw^®^ Ramanscope InVia (H43662 model, Gloucestershire, UK) using a green laser light source at 514 nm in the range from 500 to 3500 cm^−1^.

The electrical conductivity of composites was measured under increasing loads (up to 1500 bar), applied by a hydraulic press (Specac Atlas Manual Hydraulic Press 15T, Orpington, UK) according to Giorcelli et al. [31]. The instrument was composed of two solid copper cylinders (30 mm in diameter and 5 cm in length) and encapsulated in a hollow Plexiglas cylinder with a nominal inner diameter of 30 mm in the case of filler electrical characterization. The inner diameter was slightly higher so that it was possible to force the copper rods inside the Plexiglas cavity for the upper rod to slide inside the cylinder during the measurement. This arrangement created an internal chamber between the two cylinders where composites could be inserted, avoiding changes of measured materials when pressure was applied. Electrically insulating sheets were placed between the conductive cylinders and the load surfaces in order to ensure that the electrical current flowed through the sample. The resistance of the carbon fillers was measured using an Agilent 34401A multimeter (Keysight Technologies, Santa Rosa, CA, USA). The application of pressures over 450 bar resulted in a consistent plastic deformation of the tested samples.

The complex permittivity of the samples was measured in the GHz range by means of a cylindrical coaxial cell (EpsiMu^®^ toolkit by Multiwave Innovation, FR [32]), containing the sample as a dielectric spacer between inner and outer conductors, whose diameters are 0.3 cm and 0.7 cm, respectively, in accordance with the methodology reported by Torsello et al. [33]. Two conical parts link the cell to standard connectors, which helped to keep the characteristic impedance to 50 Ω, thus avoiding mismatch and energy loss. The suitably calibrated cell is connected to a ZVK Vector Network Analyzer (Rohde & Schwarz GMBH & Co, DE, Munich, Germany), and measurements are analyzed with a two-port transmission line technique. The electromagnetic properties of the sample are determined by de-embedding and the Nicolson–Ross–Weir transmission–reflection algorithm [34,35].

## 3. Results

### 3.1. Waste Cotton Fibers Biochar Characterization

**Figure 1:**
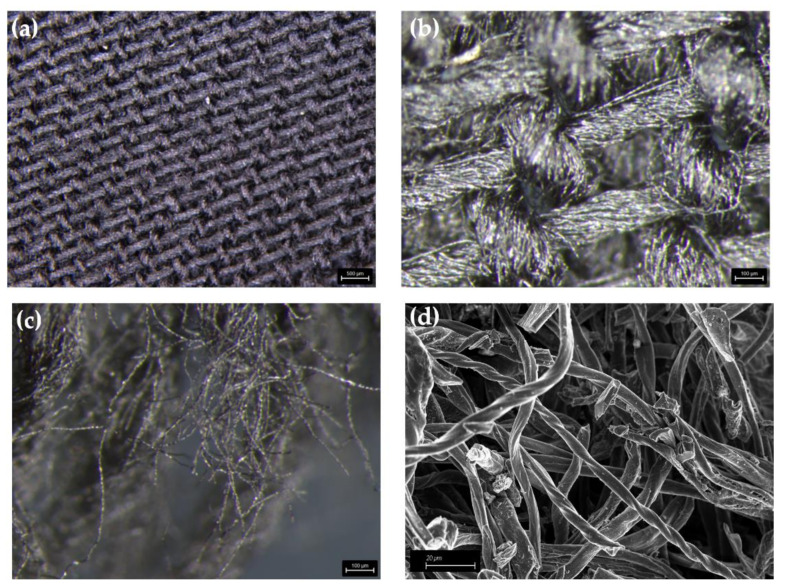
Microscopy analysis of WCB (**a**,**b**) surface and (**c**) edges prior to grinding, and (**d**) FESEM capture of WCB after grinding.

A first assessment of the morphology of WCB was carried out through optical and electron microscopy, as reported in Figure 1.

Unground WCB showed a perfect carbon replica of the original tissue, as shown in Figure 1a. The web of single WCB fibers (Figure 1b) was organized in interconnected ropes with a nominal diameter of up to around 100 μm. Across the edges of unground WCB, we observed poorly interconnected fibers with a random organization (Figure 1c). Grinded WCB fibers (Figure 1d) were characterized by a diameter of up to around 5 μm and a length of up to several hundreds of microns.

**Figure 2:**
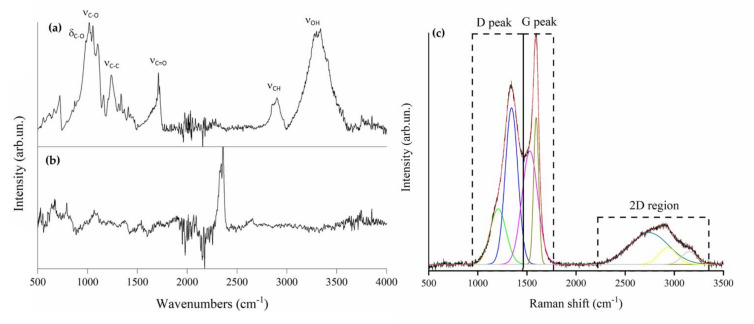
Spectroscopic analysis of neat cotton fibers and WC. FT-IR (ATR mode) spectra of (**a**) neat cotton fibers, (**b**) WCB, and (**c**) Raman spectrum of WCB (black line original signal, red line fitted spectrum, colored lines fitting components).

WCB carbonization was studied through spectroscopic methods, as shown in Figure 2.

The FT-IR spectrum of neat waste cotton fibers (Figure 2a) showed the broad band of ν_O-H_ (3300–3500 cm^−1^), the bands of saturated ν_C-H_ (2850–2950 cm^−1^), unsaturated δ_C-H_ (1360–1430 cm^−1^), saturated ν_C-C_ (1241 cm^−1^), ν_C-O_ (1035–1146 cm^−1^), and out-of-plane δ_O-H_ below 1010 cm^−1^. Those bands clearly identified a cellulose-derived matrix with a massive presence of polysaccharides. Interestingly, a band at around 1710 cm^−1^ (ν_C = O_), due to the carboxylic functionalities, was observed. The presence of carboxylic function could possibly be due to the bleaching process that the cotton underwent during the production of textile. This could lead to the oxidation of hydroxylic functionalities on the cellulose chains. After the pyrolytic process at 1000 °C, WCB did not show any of the characteristic bands of organic matrix, proving the accomplishment of full carbonization of waste cotton fibers.

Raman spectra of WCB (Figure 2c) showed the profiles of amorphous carbonaceous materials [36,37] with very intense D and G peaks and a less intense 2D region. D and G peaks were not totally resolved, and a considerable amount of intercomponents was appreciable, even if it was inferior compared to other lignocellulosic biochar obtained in the same conditions [31]. Accordingly, the I_D_/I_G_ ratio was attested to 1.1, suggesting the presence of a material that was still undergoing reorganization of graphitic domains into a nanocrystalline structure [36].

**Figure 3:**
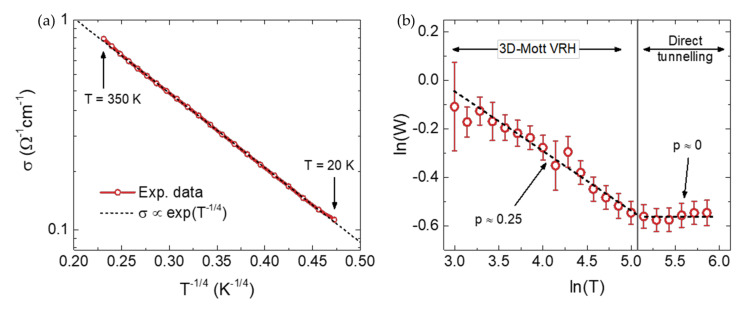
(**a**) DC electrical conductivity σ of WCB as a function of T^−1/4^ in semilogarithmic scale between 350 K and 20 K. Dashed line is the ideal scaling expected for 3D Mott variable range hopping conduction. (**b**) Reduced activation energy W=d(lnσ)/d(lnT) as a function of temperature in bilogarithmic scale. Dashed lines are linear fits to the data in the ranges where σ is dominated by 3D Mott VRH (T≤165  K) or direct tunnelling between sp^2^ domains (T≥165  K).

The temperature-dependent electrical properties of unground WCB are shown in Figure 3 between 20 and 350 K. Data points below 20 K were discarded due to a non-ideal thermal coupling between the sample and the thermometer, leaving the investigation of lower temperatures to future work.

Figure 3a shows that σ strongly decreases as the temperature is reduced from T=350 K to T=20 K. Unlike in the tea-leaf [24] biochars where σ scaled as a power law of T, here, we observe that σ scales as $\sigma \left(T\right)=\sigma_{0}\;\exp{\left(T_{0}/T\right)}^{\frac{1}{4}}$ with $T_{0}\approx 4500$ K, as highlighted by the black dashed line. According to the theory of the insulator-to-metal transition [38,39,40], such a behavior is typical of insulating materials where electric transport occurs via variable-range hopping (VRH) of the three-dimensional (3D) Mott type. This was confirmed by means of Zabrodskii analysis [41] of the reduced activation energy W=d(lnσ)/d(lnT), as shown in Figure 3b. Between $\ln\left(T\right)=3\;\left(T\approx 20\;K\right)$ and $\ln\left(T\right)=5.1\;\left(T\approx 165\;K\right)$, $\ln\left(W\right)$ linearly decreases with increasing $\ln\left(T\right)$ with a slope *p* = 0.25, perfectly consistent with 3D Mott VRH. For T≥165 K, a saturation is instead observed in the values of *W* shown in panel b, corresponding to the slight deviation in the values of σ from perfect VRH scaling observed in panel an in this higher *T* range. This suggests the occurrence of a crossover in the dominant conduction mechanism from VRH at low *T* to direct tunnelling between sp^2^ domains across sp^3^ boundaries at high *T*, as also reported in superhard carbon nanocomposites [42].

The different behavior of WCB, in comparison with tea-derived biochar [24], was possibly due to the simpler chemical structure of cotton fibers compared with lignocellulosic biomasses, which contain cellulose bonded together with lignin and hemicellulose. The pure cellulosic matrix underwent simple radical rearrangements of glucose units through dehydration processes with the formation of highly oriented aromatic clusters. This was also possible thanks to the contribution of hydrogen bonds occurring through the hydroxylic function of the cotton fibers. In the early stages of the pyrolytic conversion, hydroxylic functionalities helped the ordering process of the aromatic domains that replace the original glucose units during the carbonization process.

### 3.2. Conductivity Measurements on Composites

#### 3.2.1. DC Measurements

**Figure 4:**
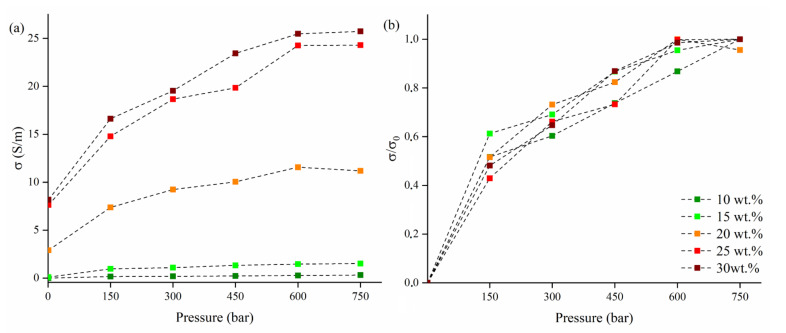
(**a**) Electrical conductivity measurement as a function of pressure WCB composites; (**b**) electrical conductivity normalized on the highest value measured across the pressure range investigated.

WCB-based composites were analyzed under the DC regime in order to evaluate the change in conductivity under pressure, as shown in Figure 4. Irreversible sample deformation was consistently observed for pressures over 450 bar [43].

The neat PVA matrix did not show any appreciable conductivity in any range of pressure analyzed. As shown in Figure 4a, WCB-containing composites filled with 10 wt.% and 15 wt.% had a very poor conductivity up to 1 and 95 mS/m without any pressure applied. Interestingly, we observed a conductivity increment up around 10 times for both composites applying 150 bar, reaching 0.159 S/m (10 wt.%) and 0.970 S/m (15 wt.%). Further pressure increments led to a quasi-linear increment of conductivity for both composites reaching 0.306 S/m (10 wt.%) and 1.52 S/m (15 wt.%) at a pressure of 750 bar. By increasing the WCB concentration, we observed an increase in conductivity up to 2.90 S/m for the composites filled with 20 wt.%, and up to 7.5 S/m for 30 wt.%. High-filled composites (20–30 wt.%) displayed an absolute increment of conductivity up to 7–16 S/m after applying 150 bar of pressure, but an inferior relative increment compared with low-filled composites (10–15 wt.%) which about doubled their initial conductivity.

As shown in Figure 4 b, the normalized conductivity shows very close trends for each curve, suggesting that the absolute value trends were mainly due to the amount of filler. The higher values of conductivity observed here, compared with previous work based on biochar produced in the same conditions, were reasonably due to both the favorable aspect ratio displayed by the deformed cylindrical morphologies [22,31,44].

#### 3.2.2. AC Measurements

**Figure 5:**
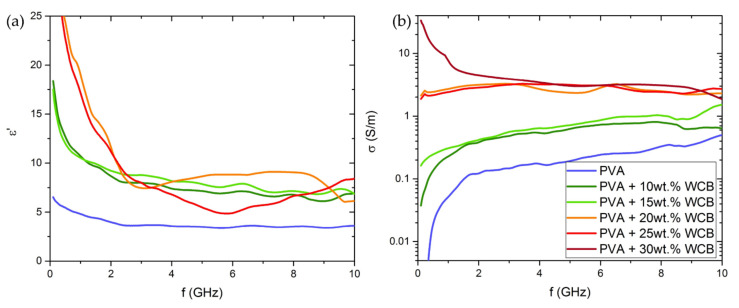
Electrical measurements as a function of frequency of WCB composites showing the trend of (**a**) complex dielectric permittivity and (**b**) electrical conductivity (logarithmic scale) from 0.1 GHz up to 10 GHz.

The high-frequency characterization of WCB-based composites is shown in Figure 5.

A monotonic increase in both the real part of the complex dielectric permittivity ε and in the AC conductivity σ with WCB loading is found for increasing filler content. The pure PVA shows negligible conductivity, slightly increasing with frequency as expected for an insulator, and a permittivity value in agreement with previous observations [45]. The 10 and 15 wt.% WCB samples show increased conductivity and permittivity, but still an insulating behavior. Starting from 20 wt.% on, the conductivity largely increases, especially at low frequency, as the material becomes conducting also in the DC limit. The high σ of the 30 wt.% sample makes the ε measurement unreliable; therefore, it is not shown in Figure 5a.

## 4. Comparison between WCB-Containing PVA Composites and Other Carbon-Based Responsive Materials

**Figure 6:**
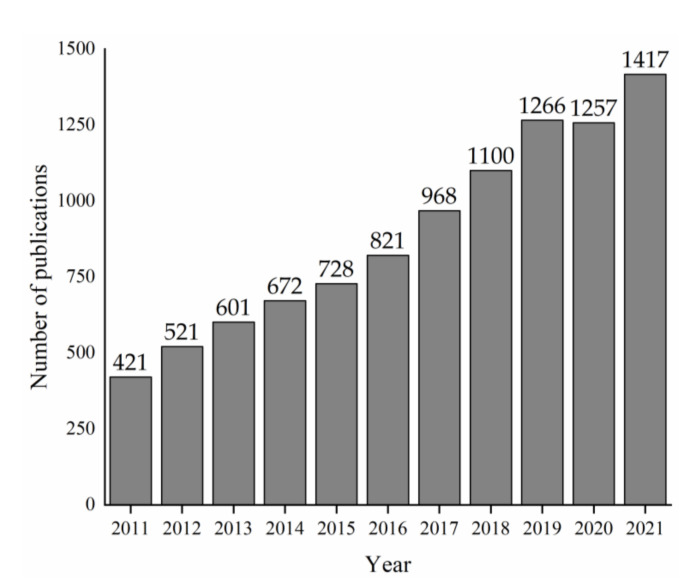
Published articles about electrically conductive composites according to the Scopus database (query “electrical conductive carbon composites”).

The use of the carbonaceous filler for the production of electrically conductive composites is a growing field, as clearly evidenced by the numerous peer-reviewed publications shown in Figure 6.

The current state of the art of electrically conductive carbon-based materials is based on the large use of nanostructured fillers, such as graphene and carbon nanotubes [46,47]. Graphene-containing PVA composites have been diffusively described by Liu et al. [48]. The authors assembled PVA nanowires together with graphene flakes, and reported the production of reversible pressure sensor with sensitivity of 28 kPa by adding only 1 wt.% of graphene. Similar results were achieved by Zhang et al. [49], combining PVA with multiwalled carbon nanotubes (MWCNT) [50]. The use of such expensive fillers helped to reach a high conductivity by adding a limited amount of material, but the high cost and environmental impact slowed down their spread towards large-scale applications. Nan et al. [51] proved that the addition of 10 wt.% of biochar to PVA helped to reach the same performances obtained by using 1 wt.% of carbon nanotubes. In this work, we extended the filler percentage to high loading, reaching conductivity values comparable with those achieved by adding carbon fibers to PVA [21] under both DC and AC regimes.

The electrical performances in the high-frequency regime reported here are consistent with those observed studying similar systems. El Shami [52] reported that the dielectric permittivity of PVA enriched with carbon quantum dots strongly decreased with increasing frequency, analogous to what we found in our WCB-based composites. By using a WCB content larger than 20 wt.% at frequencies above ~4 GHz, we achieved values of both ε’ and σ comparable with the reflection loss of PVA filled with MWCNT and polyaniline–MWCNT [53]. At low frequencies, the values of ε’ measured in WCB-filled PVA were quite similar to those reported for nanostructured fillers, such as MWCNT in the PVA matrix [54,55]. Moreover, our WCB-based composites exhibited conductivity values close to those observed for PVA composites filled with polyaniline-coated carbon fibers [56]. These evidences suggest that WCB was a valid green alternative to other common carbonaceous fillers.

Furthermore, the majority of the current literature has focused on reversible pressure-responsive carbon-based composites, whereas the development of irreversible composites has been neglected. Irreversible pressure-sensitive systems represent a valuable safety tool suitable for heavy machinery and constructions [57,58]. Plastic-based composites with irreversible piezoresistive behavior could represent a new frontier in the controlled stop of damaged mechanisms. Additionally, the WCB-based PVA composites proposed herein could be used as a coating layer to improve the MW-shielding properties of the coated material. The combination of pressure sensing, MW shielding, and environmental friendliness makes WCB-containing PVA composites a very promising multifunctional material for applications in relevant industrial sectors, such as automotive, aerospace, and construction.

## 5. Conclusions

The development of cheap, environmentally friendly, and stimuli-responsive composites is of paramount in importance in modern material science. Here we reported the development of a piezoresistive PVA composite containing WCB in concentration ranging from 10 wt.% up to 30 wt.%. By evaluating the electrical conductivity at low temperature, we observe, for the first time, hopping charge transport in unground WCB, showing the improved graphitization during the pyrolysis process. The electrical DC measurements under pressure showed two behaviors, allowing to regroup composites into poorly conductive (up to 15 wt.% of filler) and highly conductive materials (20 wt.% up). The sudden change of conductivity under plastic deformation at 150 bar is a proof of the possible utilization of these materials as irreversible piezoresistive sensors able to detect impacts. Furthermore, the AC measurements showed very attractive properties of highly loaded composites for shielding applications. Our results indicated that these biochar–PVA composite, could represent a solid choice for the production of pressure-responsive materials with both low cost and low environmental footprint.
